# Diagnostic accuracy of ultra-low-dose CT compared to standard-dose CT for identification of non-displaced fractures of the shoulder, knee, ankle, and wrist

**DOI:** 10.1186/s13244-023-01389-7

**Published:** 2023-03-08

**Authors:** Mengqiang Xiao, Meng Zhang, Ming Lei, Fenghuan Lin, Yanxia Chen, Jun Chen, Jinfeng Liu, Jingzhi Ye

**Affiliations:** Zhuhai Hospital, Guangdong Provincial Hospital of Traditional Chinese Medicine, 53 Jingle Road, Zhuhai City, Guangdong Province China

**Keywords:** Fractures, Bones, Tomography (X-ray computed), Ultra-low-dose

## Abstract

**Objectives:**

To compare the performance of ultra-low-dose computed tomography (ULD-CT) with standard-dose computed tomography (SD-CT) for the diagnosis of non-displaced fractures of the shoulder, knee, ankle, and wrist.

**Methods:**

This prospective study enrolled 92 patients receiving conservative treatment for limb joint fractures who underwent SD-CT followed by ULD-CT at a mean interval of 8.85 ± 1.98 days. Fractures were characterized as displaced or non-displaced. Objective (signal-to-noise ratio, contrast-to-noise ratio) and subjective CT image quality were evaluated. Observer performance for ULD-CT and SD-CT detecting non-displaced fractures was estimated by calculating the area under the receiver operating characteristic (ROC) curve (*A*_*z*_).

**Results:**

The effective dose (ED) for the ULD-CT protocol was significantly lower than the ED for the SD-CT protocol (*F* = 422.21~2112.25, *p* < 0.0001); 56 patients (65 fractured bones) had displaced fractures, and 36 patients (43 fractured bones) had non-displaced fractures. Two non-displaced fractures were missed by SD-CT. Four non-displaced fractures were missed by ULD-CT. Objective and subjective CT image quality was significantly improved for SD-CT compared to ULD-CT. The sensitivity, specificity, PPV, NPV, and diagnostic accuracy of SD-CT and ULD-CT for non-displaced fractures of the shoulder, knee, ankle and wrist were similar: 95.35% and 90.70%; 100% and 100%; 100% and 100%; 99.72% and 99.44%; and 99.74% and 99.47%, respectively. The *A*_*z*_ was 0.98 for SD-CT and 0.95 for ULD-CT (*p* = 0.32).

**Conclusion:**

ULD-CT has utility for the diagnosis of non-displaced fractures of the shoulder, knee, ankle, and wrist and can support clinical decision-making.

## Key points

1. ULD-CT qualified the requirements for diagnosis of non-displaced fractures.
2. ULD-CT for limb joint fractures is a useful alternative to digital radiography.
3. ULD-CT protocol for limb joint imaging effectively reduces the dose of radiation.

## Background

Fracture of the bones that make up the joints of an upper or lower extremity is a common acute health issue. Computed tomography (CT) is the gold-standard imaging modality for the diagnosis and evaluation of limb joint fractures [1] and is used for clinical decision-making [2–4]. In 2019, in the USA, an estimated 90 million CT scans were performed [5].

Standard-dose CT (SD-CT) is associated with 70–100 times the radiation exposure of conventional X-rays, and medical radiation from CT scans is responsible for approximately 0.4% of all malignant tumors [6]. Increasingly, CT scans for fractures are optimized to reduce radiation doses to “as low as reasonably achievable” while maintaining diagnostic accuracy [8–10].

Although low-dose (LD-CT) and ultra-low-dose CT (ULD-CT) (effective dose [ED] 0.53–900 μSv) have been used to diagnose selected limb fractures [7, 9, 11, 12], reports on the use of ULD-CT for the diagnosis of non-displaced limb fractures are scarce. The objective of this study was to compare the performance of ULD-CT with SD-CT for the diagnosis of non-displaced fractures of the shoulder, knee, ankle, and wrist.

## Methods

### Study population

This prospective study enrolled patients receiving conservative treatment for musculoskeletal complaints between November 30, 2019, and April 25, 2021. Inclusion criteria were (1) age ≥ 18 years; (2) recent history of trauma; (3) diagnosis of fracture on digital radiography (DR) or suspected fracture; and (4) clinical indication for SD-CT. Exclusion criteria were (1) metal implant; (2) history of tumors; or (3) history of arthritis or bone metabolic disease.

Included patients underwent clinically indicated SD-CT followed by an ULD-CT at an interval of 1–2 weeks. CT re-examination was required for non-operated fracture patients within 2 weeks to evaluate any increase in the degree of fracture displacement necessitating surgery [13].

The protocol for this study was approved by the Ethics Committee of  Guangdong Provincal Hospital of Traditional Chinese Medicine (BF2019-030-01). All patients provided written informed consent for the acquisition of a ULD-CT after a SD-CT.

### Scan protocols

Scans were performed using a Canon 320-detector-row CT scanner (Aquilion One Vision; Canon Medical Systems, Otawara, Japan). For the shoulder, knee, ankle, and wrist, SD-CT scanning parameters were 120 kV tube voltage and 150, 120, 120, and 50 mA tube current, respectively; ULD-CT scanning parameters were 80 kV tube voltage and 52, 11, 11, and 4 mA tube current, respectively; scan range was 160 mm, 140 mm, 140 mm, and 100 mm, respectively. Scan slice thickness was 0.5–1 mm. CTDIvol (mGy) and DLP (mGy*cm) were automatically implemented for all CT-protocols by the scanner software.

Effective dose (ED = DLP*k) for each patient was calculated by multiplying DLP by *k* (a conversion coefficient): shoulder *k* = 0.0113 (SD-CT) *k* = 0.0091 (ULD-CT); knee *k* = 0.0004 (SD-CT and ULD-CT); ankle and wrist *k* = 0.0002 (SD-CT and ULD-CT) [12]

Post-processing was performed on a dedicated workstation (VitreaFX3.0). Image reconstruction involved multiplanar reformatting (MPR), volume rendering (VR), and maximum intensity projection (MIP).

### Image evaluation

Two senior clinicians with 10–13 years of experience in musculoskeletal diseases independently reviewed each image to characterize each fracture as displaced or non-displaced. Displaced fractures were defined as having a fracture line > 2 mm wide and/or > 1 mm displacement of the bone cortex. Non-displaced fractures were defined as having no angulation or shortening, a fracture line < 2 mm wide, and/or < 1 mm displacement of the bone cortex [14–16]. Avulsion fractures caused by a sudden and violent pull of a muscle or ligament were characterized as displaced or non-displaced fractures when bone fragment displacement was > 5 mm or < 5 mm, respectively [16]. Each clinician reviewed each image twice at an interval of > 6 weeks. Disagreements about image interpretation were resolved through discussion and consensus.

A final diagnosis was made based on the CT/DR review within 1–3 months based on the presence of a callus at the fracture end, dysplasia, and an old fracture without a callus [8, 16]^.^

One experienced radiologist evaluated objective CT image quality metrics. A region of interest (ROI) (70 mm^2^) was placed within the muscles around the joints. Mean/standard deviation CT values of muscle (CTm) were determined from three measurements. A ROI (8 mm^2^) was placed on the thickest region of the cross section of the cortical shell of the bones of the joint. Mean/standard deviation CT values of bone (CTb) were determined from three measurements. CT values of joint cortical bone (CTc) were calculated as: CTb-CTm. Noise was calculated as mean CTm standard deviation. Signal-to-noise ratio (SNR) was calculated as: mean CTm/mean CTm standard deviation. Contrast-to-noise ratio (CNR) was calculated as (mean CTc–mean CTm) /mean CTm standard deviation [16].

Two experienced radiologists and two orthopedic physicians evaluated subjective CT image quality and the impact of subjective CT image quality on clinical decision-making on a 5-point Likert-type scale (Table 1).

**Table 1:** 5-point Likert-type scale evaluating subjective CT image quality and impact of subjective CT image quality on clinical decision-making

Scoring criteria	Subjective image quality	Impact of image quality on clinical decision-making
5	Excellent visualization of fracture line; no influence on fracture diagnosis	Excellent definition of fracture line and fracture displacement; no influence on clinical decision-making
4	Good visualization of fracture line; no influence on fracture diagnosis	Good definition of fracture line and fracture displacement; no influence on clinical decision-making
3	Adequate visualization of fracture line; no influence on fracture diagnosis	Adequate definition of fracture line and fracture displacement; no influence on clinical decision-making
2	Poor visualization of fracture line; greatly impacts fracture diagnosis	Poor definition of fracture line and fracture displacement; impacts clinical decision-making
1	Extremely poor visualization of fracture line; diagnosis is difficult or impossible	Extremely poor definition of fracture line and fracture displacement; impacts clinical decision-making

### Statistical analysis

Statistical analyses were conducted using IBM SPSS Statistics, v26.0 (IBM Corp., Armonk, NY, USA). CTDIvol, DLP, ED, CTc, SNR, and CNR for SD-CT and ULD-CT were compared using analysis of variance (ANOVA), or Tamhane's T2 test for data with unequal variances. Subjective CT image quality and the impact of subjective CT image quality on clinical decision-making for SD-CT and ULD-CT were compared with the rank sum test. The consistency of the two radiologists on the 5-point Likert-type scale evaluating subjective CT image quality and the impact of subjective CT image quality on clinical decision-making was assessed using the intraclass correlation coefficient (ICC), where < 0.40 = poor consistency; 0.41–0.60 = moderately consistent; 0.61–0.80 = good consistency; 0.81–1.00 = perfect consistency. The sensitivity, specificity, positive predictive value (PPV), negative predictive value (NPV), and accuracy of SD-CT and ULD-CT for the diagnosis of non-displaced fractures of the shoulder, knee, ankle, and wrist were calculated. Observer performance for ULD-CT and SD-CT was estimated by calculating the area under the Receiver Operating Characteristic (ROC) curve (*A*_*z*_).

## Results

**Fig. 1:**
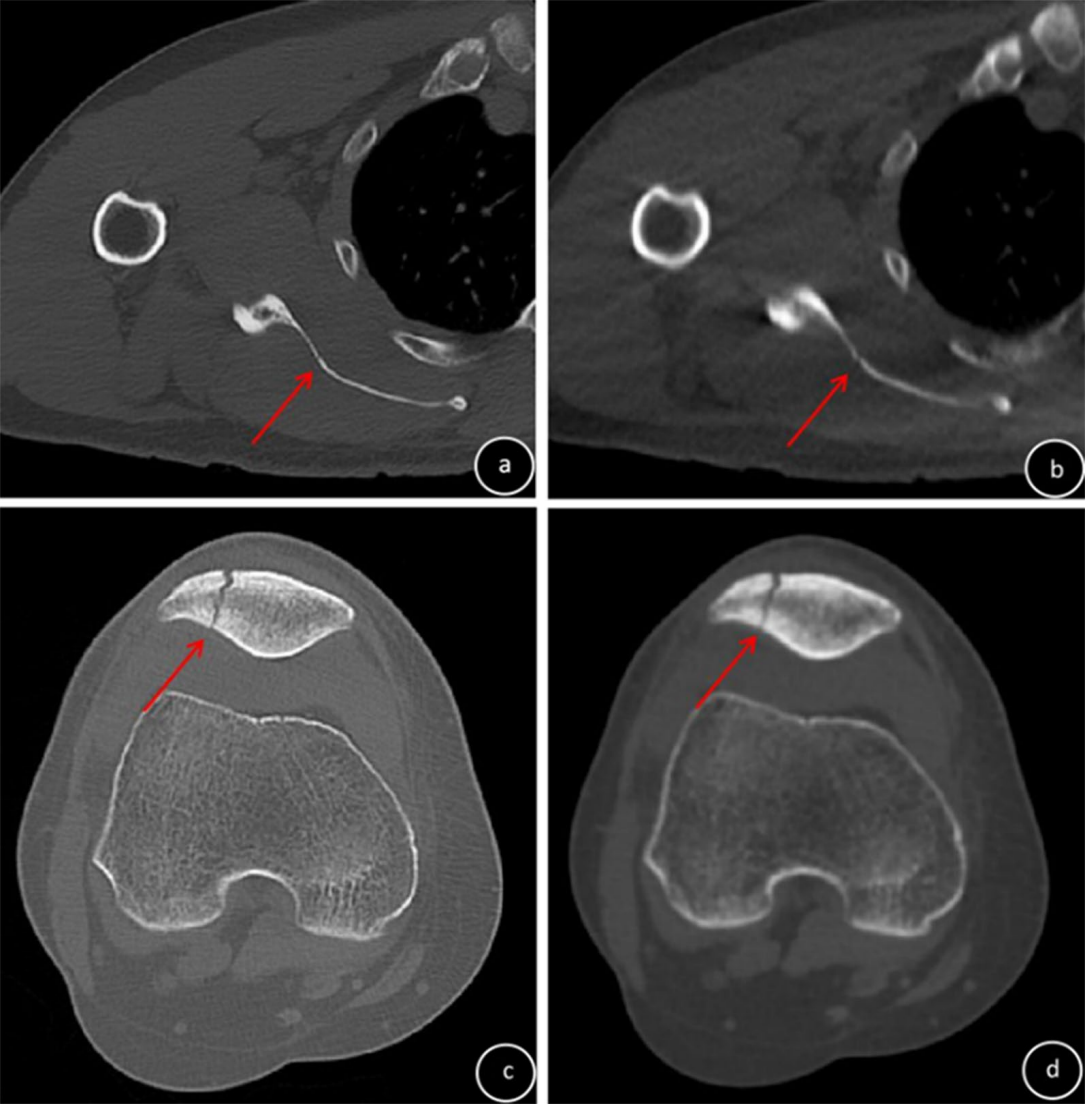
Diagnosis of non-displaced fracture of the shoulder (**a**, **b**) or knee (**c**, **d**) (red arrow). **a** and **c**: SD-CT images (subjective CT image quality and impact of subjective CT image quality on clinical decision-making, 5 points); **b** and **d** ULD-CT images (subjective CT image quality and impact of subjective CT image quality on clinical decision-making, 3 points)

**Fig. 2:**
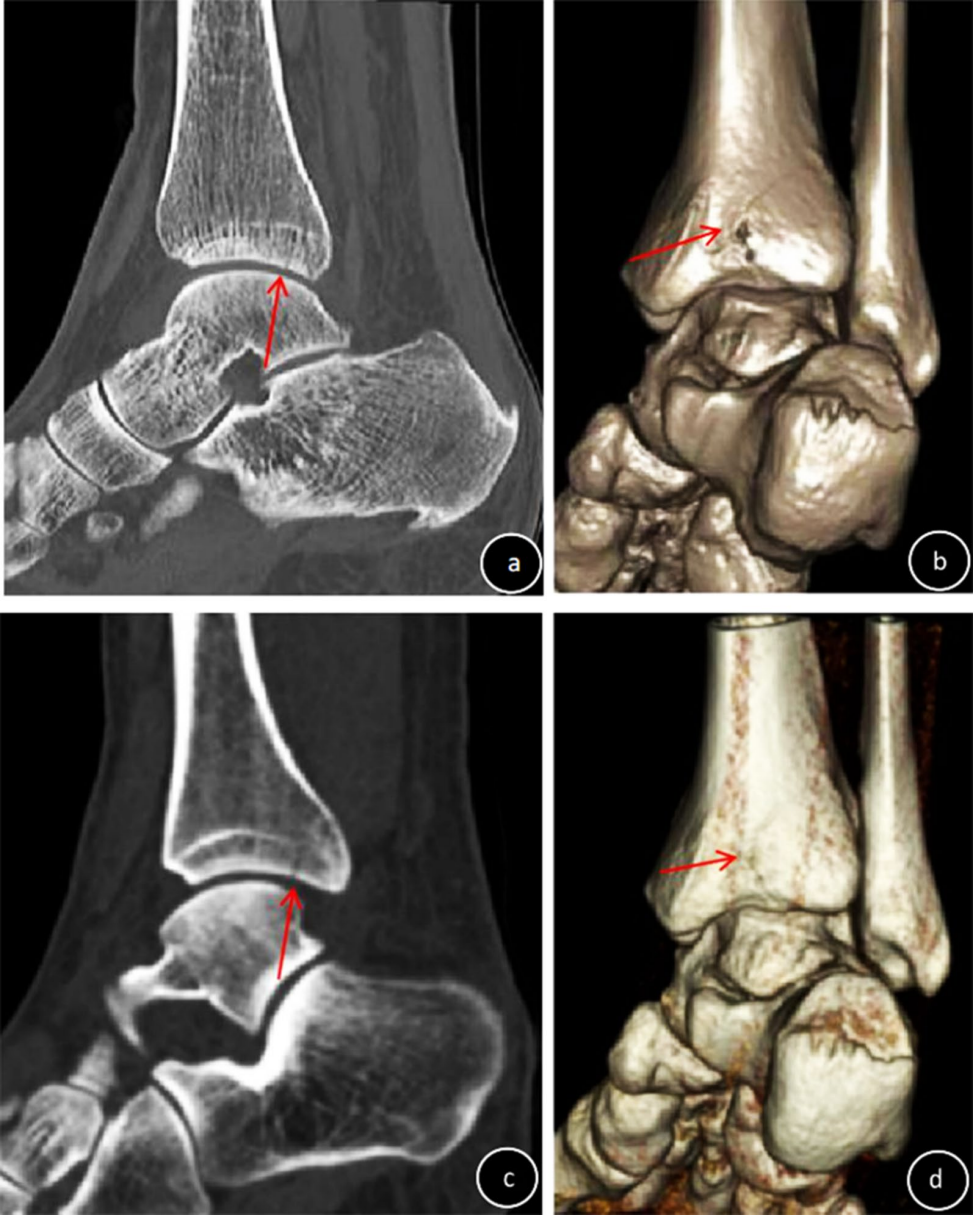
Missed diagnosis of non-displaced fracture of the ankle on SD-CT and ULD-CT (the fracture line can be seen as the red arrow). **a** SD-CT image (subjective CT image quality and impact of subjective CT image quality on clinical decision-making, 5 points); **b** SD-CT three-dimensional reconstruction; **c** ULD-CT image (subjective CT image quality, 4 points, impact of subjective CT image quality on clinical decision-making, 5 points); **d** ULD-CT three-dimensional reconstruction

**Fig. 3:**
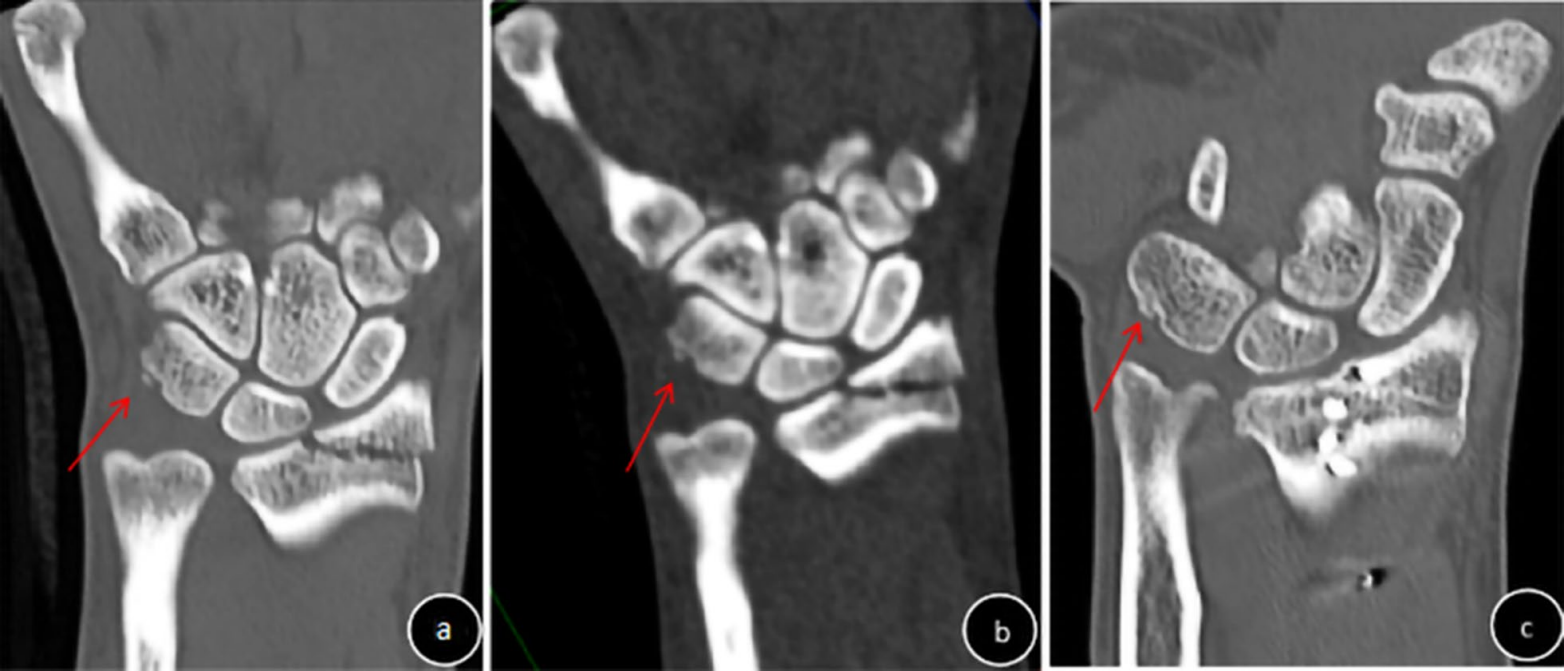
Triangular avulsion fracture of the wrist (red arrow). **a** SD-CT image; **b** ULD-CT image; **c** 3-month follow-up. SD-CT scan diagnosed an old fracture. ULD-CT scan diagnosed an avulsion fracture

This study included 92 patients receiving conservative treatment for fractures of the shoulder, knee, ankle, or wrist who underwent SD-CT and ULD-CT at an interval of 1–2 weeks (mean, 8.85 ± 1.98 days); 24 patients (72 bones) had shoulder fractures, 17 patients (68 bones) had knee fractures, 25 patients (225 bones) had ankle fractures, and 26 patients (390 bones) had wrist fractures. Of these, 56 patients (65 fractured bones; 17 fractured shoulders, 14 fractured knees, 10 fractured ankles and 6 fractured wrists, with 1 bone fracture per joint; 1 fractured ankle and 8 fractured wrists, with 2 bone fractures per joint) had displaced fractures, and 36 patients (43 fractured bones; 7 fractured shoulders (Fig. 1a, b), 3 fractured knees (Fig. 1c, d), 12 fractured ankles and 7 fractured wrists, with 1 bone fracture per joint; 2 fractured ankles and 5 fractured wrists, with 2 bone fractures per joint (Figs. 2 , 3) had non-displaced fractures. Four non-displaced fractures were missed by ULD-CT (Fig. 2).

The ED for the ULD-CT protocol was significantly lower (shoulder, knee, ankle, wrist: 23.21 ± 2.24 μSv, 2.10 ± 0.25 μSv, 1.02 ± 0.11, 0.29 ± 0.06 μSv, respectively) than the ED for the SD-CT protocol (shoulder, knee, ankle, wrist: 2513.49 ± 345.13 μSv, 71.05 ± 8.57μSv, 34.60 ± 3.65 μSv, 9.40 ± 2.26 μSv, respectively) (F = 422.21~2112.25, *p* < 0.0001; Table 2).

**Table 2:** Comparison of ULD-CT and SD-CT protocols

Group	Shoulder (*n* = 24)			Knee (*n* = 17)			Ankle (*n* = 25)			Wrist (*n* = 26)		
	CTDI (mGy)	DLP (mGy × cm)	ED (μSv)	CTDI (mGy)	DLP (mGy × cm)	ED (μSv)	CTDI (mGy)	DLP (mGy × cm)	ED (μSv)	CTDI (mGy)	DLP (mGy × cm)	ED (μSv)
SD	14.78 ± 0.65	222.43 ± 30.54	2513.49 ± 345.13	11.46 ± 0.74	177.62 ± 21.43	71.05 ± 8.57	11.29 ± 0.39	173.01 ± 18.26	34.60 ± 3.65	4.43 ± 0.36	47.01 ± 11.30	9.40 ± 2.26
ULD	1.66 ± 0.06	25.51 ± 2.47	23.21 ± 2.24	0.34 ± 0.05	5.25 ± 0.61	2.10 ± 0.25	0.34 ± 0.05	5.82 ± 0.54	1.02 ± 0.11	0.10 ± 0.00	1.47 ± 0.32	0.29 ± 0.06
F	9827.75	991.24	1249.48	3833.18	1098.36	1098.36	19,564.92	2112.25	2112.25	3837.55	422.21	422.21
P	< 0.0001	< 0.0001	< 0.0001	< 0.0001	< 0.0001	< 0.0001	< 0.0001	< 0.0001	< 0.0001	< 0.0001	< 0.0001	< 0.0001

Objective CT image quality (noise, SNR, CNR) was significantly improved for SD-CT compared to ULD-CT (*F* = 16.42~1808.07, *p* < 0.0001). CTc was significantly higher for ULD-CT compared to SD-CT (*F* = 23.97~136.48, *p* < 0.0001; Table 3) Scores for subjective CT image quality and the impact of subjective CT image quality on clinical decision-making were ≥ 3 points for SD-CT and ULD-CT, but were significantly improved for SD-CT compared to ULD-CT (Z = − (3.76–4.46), *p* < 0.0001) (Figs. 1, 2, 3, Table 4). ICC values for subjective CT image quality and the impact of subjective CT image quality on clinical decision-making for SD-CT and ULD-CT indicated good consistency (ICC = 0.65–0.99, Table 4).

**Table 3:** Comparison of objective CT image quality between ULD-CT and SD-CT

Group	Shoulder (*n* = 24)				Knee (*n* = 17)			
	Noise	SNR	CNR	CTc	Noise	SNR	CNR	CTc
SD	48.52 ± 9.20	1.53 ± 0.96	26.32 ± 15.06	1669.88 ± 217.98	70.88 ± 13.19	1.39 ± 0.45	32.80 ± 17.58	2028.50 ± 132.08
ULD	138.42 ± 40.00	0.51 ± 0.23	15.89 ± 6.41	2038.06 ± 287.64	154.13 ± 32.21	0.57 ± 0.11	15.29 ± 2.91	2374.52 ± 260.04
F	115.14	25.79	44.10	24.98	97.68	52.74	16.42	23.97
P	< 0.0001	< 0.0001	< 0.0001	< 0.0001	< 0.0001	< 0.0001	< 0.0001	< 0.0001

Group	Ankle (*n* = 25)				Wrist (*n* = 26)			
	Noise	SNR	CNR	CTc	Noise	SNR	CNR	CTc
SD	41.39 ± 10.03	2.54 ± 0.78	54.39 ± 19.74	2223.96 ± 321.96	41.15 ± 8.42	2.34 ± 0.51	34.49 ± 22.23	2888.46 ± 184.09
ULD	162.49 ± 10.11	0.60 ± 0.05	16.64 ± 2.16	2798.98 ± 365.96	189.89 ± 84.25	0.53 ± 10	15.73 ± 2.87	1926.76 ± 377.23
F	1808.07	161.89	90.37	34.79	80.20	42.73	200.46	136.48
P	< 0.0001	< 0.0001	< 0.0001	< 0.0001	< 0.0001	< 0.0001	< 0.0001	< 0.0001

**Table 4:** Comparison of subjective CT image quality and the impact of subjective CT image quality on clinical decision-making between ULD-CT and SD-CT

Fracture		Subjective evaluation				Impact of image quality on clinical decision-making			
		SD	ULD	Z	P	SD	ULD	Z	P
Shoulder (*n* = 24)	Score	4.99 ± 0.01	3.17 ± 0.38	− 4.61	< 0.0001	4.99 ± 0.01	3.33 ± 0.48	− 4.46	< 0.0001
	ICC	0.99	0.65			0.99	0.61		
Knee (*n* = 17)	Score	4.99 ± 0.01	3.06 ± 0.24	− 4.12	< 0.0001	4.99 ± 0.01	3.23 ± 0.44	− 3.83	< 0.0001
	ICC	0.99	0.72			0.99	0.75		
Ankle (*n* = 25)	Score	4.99 ± 0.01	4,16 ± 0.69	− 3.83	< 0.0001	4.99 ± 0.01	4.24 ± 0.66	− 3.76	< 0.0001
	ICC	0.99	0.96			0.99	0.95		
Wrist (*n* = 26)	Score	4.99 ± 0.01	3.19 ± 0.40	− 4.78	< 0.0001	4.99 ± 0.01	3.45 ± 0.63	− 4.66	< 0.0001
	ICC	0.99	0.75			0.99	0.90		

The sensitivity, specificity, PPV, NPV, and diagnostic accuracy of SD-CT and ULD-CT for non-displaced fractures of the shoulder, knee, ankle, and wrist were similar: 95.35% and 90.70%; 100% and 100%; 100% and 100%; 99.72% and 99.44%; and 99.74% and 99.47%, respectively.

The *A*_*z*_ value for detecting non-displaced fractures using SD-CT was 0.98 (95%CI: 0.96–0.99), which was slightly higher than ULD-CT (0.95, [95%CI: 0.94–0.97], *p* = 0.32).

## Discussion

This study compared the performance of ULD-CT with SD-CT for the diagnosis of non-displaced fractures of the shoulder, knee, ankle, and wrist. Findings showed the ED for the ULD-CT protocol was significantly lower than the ED for the SD-CT protocol and lower than that for DR [17]. ULD-CT image quality was inferior to SD-CT, but the sensitivity, specificity, PPV, NPV, and diagnostic accuracy of SD-CT and ULD-CT for non-displaced fractures of the shoulder, knee, ankle, and wrist were similar. To the author’s knowledge, this is the first study to show that ULD-CT is a feasible alternative to SD-CT for imaging these types of fracture.

Non-displaced fractures are challenging to detect by physical examination and DR [18]. Missed or misdiagnosis of a non-displaced fracture can delay treatment and have a negative impact on patients' functional recovery [18]. Reports on the diagnostic performance of CT for assessment of non-displaced fractures are scarce [19, 20], potentially due to the lack of a clear definition of displaced versus non-displaced fractures [14–16]. A fracture is considered non-displaced if the fragments remain aligned. Displaced fragments are defined as the abnormal position of the distal fracture fragment in relation to the proximal bone and may be minimal, moderate, or severe. Non-displaced or minimally displaced fractures can be difficult to differentiate, but it is important to distinguish between these as treatment and outcome can vary [21].

Previous studies have assessed the clinical utility of ULD-CT for evaluating select limb fractures [7, 9, 11–13, 16]. Two studies showed ULD-CT (tube voltage, 80–120 kV; tube current, 10 mA) was a useful alternative to DR in the evaluation of acute wrist and ankle fractures [9, 22]. In another study, a ULD-CT protocol (tube voltage, 120 kV; tube current, 15–19 mA) that decreased the ED by a factor of 14 compared to SD-CT provided high-quality images for reliable detection of various types of limb fracture identifiable on screening plain radiographs of an injured body part [20]. In the present study, scanning parameters were designed to reduce ED according to the size and thickness of limb joints. The highest ED was used for the shoulder (80 kv, 52 mAs). The lowest ED (80 kV, 5 mAs) was used for the wrist. In a previous study that used a similar protocol for wrist traumas in an emergency department, ULD-CT (80 kV, 5 mAs) provided high-quality images with no changes in diagnostic accuracy while reducing the radiation dose by approximately 98% compared to SD-CT [7, 23].

The present study was associated with several limitations. First, the sample size was small. Second, SD-CT and ULD-CT were performed at an interval of 1–2 weeks; during this time, fractures may have changed. Third, the ULD-CT protocol was not compared to DR or magnetic resonance imaging (MRI). Finally, the impact of SD-CT and ULD-CT on perioperative outcomes was not evaluated.

## Conclusion

The sensitivity, specificity, and diagnostic accuracy of SD-CT and ULD-CT for non-displaced fractures of the shoulder, knee, ankle, and wrist were similar. These data imply that ULD-CT has utility for the diagnosis of non-displaced fractures of the shoulder, knee, ankle and wrist, and can support clinical decision-making.

## Data Availability

The datasets used or analyzed during the current study are available from the corresponding author on reasonable request.

## Abbreviations

CNR: Contrast-to-noise ratio.
CTb: CT values of bone
CTm: CT values of muscle
DR: Digital radiography
ED: Effective dose
MIP: Maximum intensity projection
MPR: Multiplanar reformatting
ROC: Receiver operating characteristic
ROI: Region of interest
SD-CT: Standard-dose computed tomography
SNR: Signal-to-noise ratio
ULD-CT: Ultra-low-dose computed tomography
VR: Volume rendering
